# Treatment of Colic

**Published:** 1880-01

**Authors:** 


### TREATMENT OF COLIC.

Phares' method consists in inversion, or simply turning the patient upside down. Colic of several days duration is so relieved in a few minutes. The elbow knee position or laying on the face, head, and shoulders hanging down over the side of the bed may answer; complete inversion however is the best.—*Atlanta Medical and Surgical Journal.*

